# Development and Validation of the Shorter Versions of the Patient Care Ownership Scale for Use in Japan

**DOI:** 10.31662/jmaj.2026-0093

**Published:** 2026-07-10

**Authors:** Hirohisa Fujikawa, Kentaro Sakamaki, Hidetaka Tamune, Yuji Nishizaki, Hiroyuki Kobayashi, Yasuharu Tokuda

**Affiliations:** 1 Department of General Medicine, Juntendo University Faculty of Medicine, Tokyo, Japan; 2 Center for General Medicine Education, School of Medicine, Keio University, Tokyo, Japan; 3 Department of Medical Education Studies, International Research Center for Medical Education, Graduate School of Medicine, The University of Tokyo, Tokyo, Japan; 4 Faculty of Health Data Science, Juntendo University, Chiba, Japan; 5 Department of Psychiatry and Behavioral Science, Juntendo University Graduate School of Medicine, Tokyo, Japan; 6 Division of Medical Education, Juntendo University Faculty of Medicine, Tokyo, Japan; 7 Department of Internal Medicine, Mito Kyodo General Hospital, University of Tsukuba, Ibaraki, Japan; 8 Muribushi Okinawa for Teaching Hospitals, Okinawa, Japan; 9 Tokyo Foundation for Policy Research, Tokyo, Japan

**Keywords:** patient care ownership, professionalism, psychometric validation, scale development, medical education assessment, short version, burnout, job satisfaction

## Abstract

**Introduction:**

Effective training in patient care ownership (PCO), a key component of medical professionalism, requires reliable and valid assessment tools. Although the 13-item Japanese version of the PCO Scale (J-PCOS) is widely used, shorter instruments would aid educational initiatives to nurture PCO and facilitate further studies on PCO. Accordingly, we aimed to develop shorter versions of the J-PCOS and examine their psychometric properties.

**Methods:**

We conducted a cross-sectional study using an online anonymous survey for medical residents in Japan. We developed the following shorter versions: an 8-item version (J-PCOS-8; two from each of the four subscales), a 4-item version (J-PCOS-4; one from each of the four subscales), and a 2-item version (J-PCOS-2; one from the cognitive-behavioral aspect and one from the emotional-affective aspect). We investigated their structural validity (confirmatory factor analysis) and internal consistency reliability (Cronbach’s alpha) and examined their relations with postgraduate year (PGY), burnout, and job satisfaction.

**Results:**

A total of 1859 medical residents were included. Both the J-PCOS and J-PCOS-8 had good structural validity, with the latter exhibiting better model fit indices. Both the J-PCOS and its shorter versions demonstrated moderate to good internal consistency reliability (Cronbach’s alpha: J-PCOS, 0.95; J-PCOS-8, 0.92; J-PCOS-4, 0.86; and J-PCOS-2, 0.74). All scales showed that PGY2 residents had significantly greater PCO than PGY1 residents. They also showed that residents with burnout had lower levels of PCO than those without burnout and that residents with job satisfaction exhibited greater PCO than those without job satisfaction.

**Conclusions:**

This study suggests that the J-PCOS-8, J-PCOS-4, and J-PCOS-2 are low-burden, reliable, and valid scales for measuring PCO. These shorter versions may allow researchers to reduce questionnaire length, potentially resulting in higher response rates, improved completion rates, and better data quality, while enabling clinical educators to use them in daily educational initiatives.

## Introduction

Patient care ownership (PCO) is a concept that embodies a number of professionalism attributes and has recently received considerable attention in the medical education literature ^(1), (2)^. The term refers to the cognitive-affective state that emerges as a consequence of the individual’s knowledge of, management of, and emotional investment in a relationship with patients ^(3)^. The concept of PCO is multifaceted, encompassing physician behaviors (e.g., advocacy for patients), physician attitudes (e.g., going above and beyond the minimum required), physician identity (e.g., assuming the role of a main care provider), physician qualities (e.g., taking initiative), and quality of patient care (e.g., providing best care) ^(4)^. A recent study indicated that greater PCO was negatively associated with burnout and was positively associated with job satisfaction ^(5)^. Moreover, higher levels of PCO may contribute to better clinical skills, improved patient care, and higher patient satisfaction ^(3), (6)^. Given these potential benefits, understanding and assessing PCO among physicians, particularly during training ^(6), (7)^, has become a crucial training and research focus.

To ensure effective education and research initiatives regarding PCO, it is essential to have a validated assessment scale. The PCO Scale (PCOS) for medical residents, developed by Djulbegovic et al. ^(8), (9)^, is a validated and widely used measure that fulfills this requirement. A Japanese version of the PCOS (J-PCOS) has been developed, and its psychometric properties have been well examined ^(10)^. The development of the J-PCOS has facilitated research on PCO in Japan ^(5), (11), (12), (13)^, providing insights into its role in medical training and its potential impact on physician well-being and patient outcomes.

Despite the value of the J-PCOS, its 13-item length poses challenges for practical use in research and educational settings. Lengthy questionnaires can contribute to challenging demands placed on respondents and thus lower response rates ^(14)^. To enhance the usability of the scale, developing shorter versions of the J-PCOS is necessary. In particular, medical residents are usually busy with a number of tasks (e.g., direct patient care, documentation, and communication with other healthcare staff) ^(15), (16)^ and have limited time to complete questionnaires during working hours. Considering the implementation of physician working hour regulations in Japan in April 2024 ^(17), (18)^, it is plausible that the time available for completing surveys is becoming increasingly limited. Against this background, there is increasing pressure on researchers to develop reliable, valid, and short instruments for measuring PCO among medical residents.

Accordingly, the aim of this study was to develop and validate shorter versions of the J-PCOS to improve its feasibility for educational and research use while reducing respondent burden. In practice, the optimal scale length may vary depending on the intended application. Therefore, we sought to develop multiple shorter versions with different levels of brevity to accommodate these varying needs. Specifically, we developed 8-item, 4-item, and 2-item versions of the J-PCOS, assuming that each would serve a distinct purpose: the 8-item version for relatively detailed assessment, the 4-item version for rapid screening, and the 2-item version for ultra-brief assessment in large-scale surveys. We hypothesized that the shorter versions would demonstrate acceptable psychometric properties comparable to those of the original 13-item scale while retaining theoretically meaningful associations with training stage and well-being–related variables (as described in detail in the following sections). The development of shorter versions of the scale may facilitate further educational initiatives focused on nurturing PCO and more large-scale research into PCO, which could in turn contribute to more substantial PCO education and ultimately to enhanced quality of patient care.

## Materials and Methods

### Study design, setting, and participants

This nationwide cross-sectional study formed one component of a research project on PCO between January 17 and March 31, 2024. We distributed an online anonymous survey to medical residents in postgraduate year 1 (PGY1) and PGY2 in Japan who took the General Medicine In-Training Examination (GM-ITE). The GM-ITE is a nationwide examination developed by the Japan Institute for Advancement of Medical Education Program (JAMEP), a nonprofit organization, and is now taken by over half of Japanese medical residents ^(13), (19), (20)^. The examinees were asked to participate in our study immediately after the GM-ITE. Prior to participating in the study, they read a research explanation document that informed them of the anonymous and voluntary nature of the study. We included only examinees who consented to participate in the study.

### The J-PCOS

The J-PCOS includes 13 items. Factor analysis identified a four-factor structure: assertiveness (6 items), sense of ownership (2 items), diligence (2 items), and being the “go-to” person (3 items) ^(10)^. A recent study suggested that assertiveness and being the “go-to” person can be categorized as cognitive-behavioral aspects of PCO, while sense of ownership and diligence can be classified as emotional-affective aspects (Table 1) ^(21)^. Assertiveness is described as the ability to communicate with confidence and clarity regarding one’s thoughts, feelings, and needs in relation to patient care. It enables physicians to advocate for their patients, express concerns, and collaborate with their colleagues ^(2), (4)^. Sense of ownership reflects a physician’s perceived commitment to their patients and their care ^(2), (4)^. Diligence involves a strong work ethic, attention to detail, and perseverance in patient care ^(2), (4)^. Being the “go-to” person means being a reliable source of information and support for both patients and colleagues. This role involves having in-depth knowledge of patient conditions, therapy, and resources, as well as being approachable and willing to assist others ^(2), (4)^.

**Table 1.** The J-PCOS and its Shorter Versions.

Factors (aspects)	Items	Item-total correlation	J-PCOS-8	J-PCOS-4	J-PCOS-2
Assertiveness (cognitive-behavioral)	Q1. I was vocal and assertive about my patients’ best treatment/care.	0.763			
	Q4. I ensured good continuity of care even when I was absent from the service.	0.802			
	Q5. I felt comfortable telling the attending what I felt was the right thing to do for my patients, rather than just letting them decide.	0.811	Included		
	Q7. I was given the opportunity to make decisions independently about my patients’ care.	0.755			
	Q10. I felt comfortable making decisions independently about my patients’ care.	0.823	Included	Included	Included
	Q11. I challenged the team as needed if I felt it was in my patients’ best interest, no matter how much push back I got.	0.807			
Sense of ownership (emotional-affective)	Q12. I felt responsible for my patients’ care, even after my shift ended.	0.728	Included		
	Q13. I felt a strong sense of ownership of my patients’ care.	0.788	Included	Included	Included
Diligence (emotional-affective)	Q8. I personally made sure to go back and check that all orders were actually carried out.	0.781	Included	Included	
	Q9. When carrying out my patient’s management plan, I took extra care to make sure that things did not fall through the cracks.	0.772	Included		
Being the “go-to” person (cognitive-behavioral)	Q2. I was the “go-to” person for knowledge about my patients.	0.818	Included	Included	
	Q3. I was proactive in checking up on my patients, rather than being called with questions or concerns.	0.810	Included		
	Q6. I made sure that the nursing staff was updated with the day’s plan.	0.805			

J-PCOS: Japanese version of the Patient Care Ownership Scale; J-PCOS-2: 2-item version of the J-PCOS; J-PCOS-4: 4-item version of the J-PCOS; J-PCOS-8: 8-item version of the J-PCOS.

All items were rated on a 7-point Likert scale, with response options ranging from 1 (strongly disagree) to 7 (strongly agree) ^(10)^. In this study, the score of the scale was calculated as the average of the 13 items and ranged from 1 to 7, with higher scores indicating greater PCO.

### Development of the shorter versions of the J-PCOS

We developed shorter versions of the J-PCOS using a stepwise procedure, beginning with an 8-item version (J-PCOS-8), subsequently reducing it to a 4-item version (J-PCOS-4) and finally to a 2-item version (J-PCOS-2). This reduction was based on the understanding that the J-PCOS is composed of four distinct factors and two distinct aspects, with the J-PCOS-8 encompassing 2 items per factor, the J-PCOS-4 comprising 1 item per factor, and the J-PCOS-2 including 1 item per aspect.

In the first step, we shortened the J-PCOS from 13 to 8 items using the following approach. First, the first author (Hirohisa Fujikawa), an expert on PCO who developed the J-PCOS, read the scale iteratively to familiarize himself with it. Second, Hirohisa Fujikawa checked the results of the factor analysis in the article describing the development of the J-PCOS ^(10)^, including the factor loadings of each item. Third, Hirohisa Fujikawa calculated the Pearson correlation coefficient between each item and the total score (Table 1). Fourth, Hirohisa Fujikawa chose the characteristic items of each factor based on predefined criteria (i.e., higher factor loadings, stronger item-total correlations, and conceptual representativeness of each factor) to ensure comprehensive coverage of the core elements of each factor and maintain the requisite content validity for the short version of the J-PCOS. Fifth, the item reduction process and selected items were reviewed and confirmed by all authors to ensure consistency with the predefined criteria and conceptual validity. As a result, we obtained the J-PCOS-8 (Table 1). The factor loadings of the selected items are summarized in Supplementary Table 1.

In the second step, we reduced the number of items from 8 to 4. Also at this step, we carefully reviewed the results of the factor analysis and the Pearson correlation coefficients. In addition, Hirohisa Fujikawa performed theoretical considerations of the item content. Consequently, we chose the following 4 items (Table 1): “Q10. I felt comfortable making decisions independently about my patients’ care” (assertiveness); “Q13. I felt a strong sense of ownership of my patients’ care” (sense of ownership); “Q8. I personally made sure to go back and check that all orders were actually carried out” (diligence); and “Q2. I was the ‘go-to’ person for knowledge about my patients” (being the “go-to” person). Q10 was selected on the basis of its highest Pearson correlation coefficient value, with the part “felt comfortable making decisions” being identified as the most representative of the concept of assertiveness. Q13 was selected on the basis of its higher factor loading and correlation coefficient values compared with the other item of sense of ownership, its inclusion of the key term “sense of ownership,” and its apparent embodiment of the concept of “sense of ownership.” Q8 was selected because it exhibited higher factor loadings and correlation coefficients than the other items of diligence. The phrase “personally made sure to go back and check” appeared to suggest activeness. Q2 was chosen because it demonstrated the highest values for factor loading and correlation coefficient among the items pertaining to being the “go-to” person. The phrase “being the ‘go-to’ person” was identified as the key term and appeared to embody the concept. Fifth, all authors confirmed these item choices. Thus, we developed the J-PCOS-4.

In the third step, the number of items was reduced from 4 to 2. We decided to choose 2 items, one from the cognitive-behavioral aspect (i.e., Q2 or Q10) and the second from the emotional-affective aspect (i.e., Q8 or Q13). After iterative discussions within the research team, Q10 was selected for the cognitive-behavioral aspect because it had the highest Pearson correlation coefficient value, with the part “felt comfortable making decisions” being identified as the most representative of the concept of PCO. We also decided to select Q13 for the emotional-affective aspect because it included the key term “ownership.” Thus, the J-PCOS-2 was developed. The development process of these shortened scales was reviewed and confirmed by all authors.

### Statistical analysis

Since the shorter versions are expected to perform similarly to the J-PCOS, we compared them with the J-PCOS with respect to (1) structural validity, (2) internal consistency reliability, (3) relations with PGY, and (4) relations with burnout and job satisfaction.

Confirmatory factor analysis was performed with the four-factor structure with maximum likelihood estimation. We compared the fit indices between the J-PCOS and J-PCOS-8. The indices included comparative fit index with a desired value of >0.95, root mean square error of approximation with a desired value of <0.10, and standardized root mean square residual with a desired value of <0.08 ^(22), (23)^. Because the 4-item and 2-item versions included only 1 item per domain, confirmatory factor analysis was not conducted, as the models would be under-identified. Instead, scores from these versions were interpreted as total scores based on individual items rather than as latent constructs.

To assess internal consistency reliability, we calculated Cronbach’s alpha coefficients. The values are characterized as follows: <0.50, insufficient; 0.50-0.69, moderate; 0.70-0.79, satisfactory; and 0.80 or more, good ^(24)^.

PCO is expected to be nurtured through postgraduate training, which was supported by a previous study showing that PGY was positively associated with PCO ^(11)^. Accordingly, we examined whether PGY2 residents had greater PCO, as assessed by the J-PCOS and its abbreviated forms, in comparison to PGY1 residents by employing an unpaired t-test.

Recent medical education literature on medical residents revealed that there was a negative link between PCO and burnout and a positive link between PCO and job satisfaction ^(5)^. Accordingly, in this study we investigated these links using the J-PCOS, J-PCOS-2, J-PCOS-4, and J-PCOS-8. Burnout was assessed using the Japanese version of the Single-Item Measure of Burnout Scale (SMB-J), which has good reliability and validity ^(25)^. The item is rated on a 5-point Likert scale, with response options ranging from 1 (“I feel completely burned out. I am at the point where I may need to seek help”) to 5 (“I enjoy my work. I have no symptoms of burnout”); lower scores indicate higher risk of burnout. A score of 3 or lower is defined as burnout ^(25), (26)^. We examined whether there was a significant discrepancy in PCO scores, as assessed by the J-PCOS and its shorter versions, between medical residents with burnout and those without burnout by using an unpaired t-test. Job satisfaction was evaluated using a single-item measure derived from the Mini-Z 2.0, a widely used and validated instrument of physician well-being, including its Japanese version ^(26), (27)^. This single-item measure has been employed in previous studies as a pragmatic indicator of overall job satisfaction, particularly in large-scale surveys where minimizing respondent burden is crucial ^(28), (29)^. The item (“Overall, I am satisfied with my current job”) is rated on a 5-point Likert scale, with response options ranging from 1 (strongly disagree) to 5 (strongly agree). Referring to previous literature ^(30), (31)^, we developed a dichotomous variable for job satisfaction as follows: “not satisfied” for responses of “strongly disagree,” “disagree,” or “neither agree nor disagree” and “satisfied” for responses of “agree” or “strongly agree.” We investigated whether there was a significant difference in PCO scores, as assessed by the J-PCOS and its shorter versions, between residents with job satisfaction and those without job satisfaction by employing an unpaired t-test.

We performed confirmatory factor analysis using R version 4.4.2 (The R Foundation for Statistical Computing, Vienna, Austria). All other statistical analyses were conducted using SPSS version 30.0.0.0 (IBM Corp). We chose complete case analyses. We considered a two-tailed p < 0.05 as statistically significant.

### Ethical considerations

Before participating in the study, all participants provided written informed consent by ticking the consent box in the web questionnaire. The study was approved by the ethics committee of the JAMEP (approval number: 24-10).

## Results

Of the 9106 GM-ITE examinees, 2050 expressed their willingness to participate in the study. Of these, 191 were excluded because of missing data, leaving 1859 medical residents for analysis. Table 2 shows the characteristics of the participants.

**Table 2.** Participants’ Characteristics (n = 1859).

Characteristics		Values
PGY, n (%)		
	1	915 (49.2)
	2	944 (50.8)
Sex, n (%)		
	Female	591 (31.8)
	Male	1268 (68.2)
Hospital type, n (%)		
	Community hospital	1506 (81.0)
	University main hospital	236 (12.7)
	University branch hospital	117 (6.3)
Burnout^a^, n (%)		
	1–3 (Burnout)	270 (14.5)
	4–5 (No burnout)	1589 (85.5)
Job satisfaction^b^, n (%)		
	1–3 (Not satisfied with job)	410 (22.1)
	4–5 (Satisfied with job)	1449 (77.9)
J-PCOS^c^, mean (SD)		
	Total	4.83 (0.99)
	Factor 1	4.84 (1.03)
	Factor 2	5.05 (1.09)
	Factor 3	4.89 (1.12)
	Factor 4	4.61 (1.17)
J-PCOS-8^c^, mean (SD)		
	Total	4.86 (1.00)
	Factor 1	4.82 (1.15)
	Factor 2	5.05 (1.09)
	Factor 3	4.89 (1.12)
	Factor 4	4.68 (1.19)
J-PCOS-4^d^, mean (SD)		
	Total	4.77 (1.06)
	Q10 (included in factor 1 in J-PCOS)^e^	4.71 (1.29)
	Q13 (included in factor 2 in J-PCOS)^e^	5.14 (1.10)
	Q8 (included in factor 3 in J-PCOS)	4.71 (1.32)
	Q2 (included in factor 4 in J-PCOS)	4.51 (1.37)

J-PCOS: Japanese version of the Patient Care Ownership Scale; J-PCOS-4: 4-item version of the J-PCOS; J-PCOS-8: 8-item version of the J-PCOS; PGY: postgraduate year; SD: standard deviation. ^a^Rated on a 5-point Likert scale ranging from 1 (“I feel completely burned out. I am at the point where I may need to seek help”) to 5 (“I enjoy my work. I have no symptoms of burnout”), with lower scores indicating higher risk of burnout. ^b^Rated on a 5-point Likert scale ranging from 1 (strongly disagree) to 5 (strongly agree), with higher scores indicating greater job satisfaction. ^c^The scores range from 1 to 7, with higher scores indicating greater patient care ownership. Factor 1: assertiveness; factor 2: sense of ownership; factor 3: diligence; and factor 4: being the “go-to” person. ^d^The scores range from 1 to 7, with higher scores indicating greater patient care ownership. Q10: “I felt comfortable making decisions independently about my patients’ care”; Q13: “I felt a strong sense of ownership of my patients’ care”; Q8: “I personally made sure to go back and check that all orders were actually carried out”; and Q2: “I was the ‘go-to’ person for knowledge about my patients.” ^e^These items were included in the 2-item version of the J-PCOS.

### Structural validity and internal consistency reliability

Table 3 compares the fit indices between the J-PCOS and the J-PCOS-8. All goodness-of-fit indices indicated that the J-PCOS-8 had a better fit than the original J-PCOS.

**Table 3.** Comparison of the Structural Validity and Internal Consistency Reliability between the J-PCOS and its Shorter Versions.

Scale version	CFI	RMSEA	SRMR	Cronbach’s alpha
J-PCOS^a^	0.954	0.086	0.028	0.95
J-PCOS-8^a^	0.986	0.072	0.016	0.92
J-PCOS-4^a^	N/A	N/A	N/A	0.86
J-PCOS-2^a^	N/A	N/A	N/A	0.74

CFI: comparative fit index; J-PCOS: Japanese version of the Patient Care Ownership Scale; J-PCOS-2: 2-item version of the J-PCOS; J-PCOS-4: 4-item version of the J-PCOS; J-PCOS-8: 8-item version of the J-PCOS; N/A: not applicable; RMSEA: root mean square error of approximation; SRMR: standardized root mean square residual. ^a^The scores range from 1 to 7, with higher scores indicating greater patient care ownership.

Table 3 also shows the results of the comparison of Cronbach’s alpha values between the J-PCOS and its shorter versions. While the value of Cronbach’s alpha of the J-PCOS was 0.95, the alpha values of the J-PCOS-8, J-PCOS-4, J-PCOS-2 were 0.92, 0.86, and 0.74, respectively. Since Cronbach’s alpha values increase with test length, alpha was somewhat higher for the J-PCOS than for the J-PCOS-8, for the J-PCOS-8 than for the J-PCOS-4, and for the J-PCOS-4 than for the J-PCOS-2.

### Relations with PGY

Table 4 shows the relations of scores on the J-PCOS and its shorter versions with PGY. All scales demonstrated that PGY2 residents had significantly greater PCO than PGY1 residents.

**Table 4.** Relations of Scores on the J-PCOS and its Shorter Versions with PGY.

Scale version	PGY1	PGY2	p Value^a^
J-PCOS^b^, mean (SD)	4.77 (0.99)	4.89 (0.99)	0.009
J-PCOS-8^b^, mean (SD)	4.80 (1.00)	4.91 (0.99)	0.021
J-PCOS-4^b^, mean (SD)	4.69 (1.07)	4.84 (1.05)	0.003
J-PCOS-2^b^, mean (SD)	4.87 (1.05)	4.98 (1.08)	0.036

J-PCOS: Japanese version of the Patient Care Ownership Scale; J-PCOS-2: 2-item version of the J-PCOS; J-PCOS-4: 4-item version of the J-PCOS; J-PCOS-8: 8-item version of the J-PCOS; PGY: postgraduate year; SD: standard deviation. ^a^Independent t-test. ^b^The scores range from 1 to 7, with higher scores indicating greater patient care ownership.

### Relations with burnout and job satisfaction

Table 5 shows the relations of scores on the J-PCOS and its shorter versions with burnout and job satisfaction. Examination of these scores indicated that residents with burnout had lower levels of PCO than those without burnout and that residents with job satisfaction exhibited greater PCO than those without job satisfaction. The finalized shorter versions of the J-PCOS are provided in Supplementary Items S1, S2, and S3.

**Table 5.** Relations of Scores on the J-PCOS and its Shorter Versions with Burnout and Job Satisfaction.

Relations with burnout			
Scale version	Burnout	No burnout	p Value^a^
J-PCOS^b^, mean (SD)	4.48 (1.04)	4.88 (0.97)	<0.001
J-PCOS-8^b^, mean (SD)	4.51 (1.08)	4.92 (0.97)	<0.001
J-PCOS-4^b^, mean (SD)	4.40 (1.16)	4.83 (1.03)	<0.001
J-PCOS-2^b^, mean (SD)	4.58 (1.18)	4.98 (1.04)	<0.001
**Relations with job satisfaction**			
**Scale version**	**Satisfied with job**	**Not satisfied with job**	**p Value^a^**
J-PCOS^b^, mean (SD)	4.95 (0.95)	4.38 (0.99)	<0.001
J-PCOS-8^b^, mean (SD)	4.99 (0.95)	4.40 (1.02)	<0.001
J-PCOS-4^b^, mean (SD)	4.90 (1.02)	4.29 (1.09)	<0.001
J-PCOS-2^b^, mean (SD)	5.05 (1.02)	4.48 (1.11)	<0.001

J-PCOS: Japanese version of the Patient Care Ownership Scale; J-PCOS-2: 2-item version of the J-PCOS; J-PCOS-4: 4-item version of the J-PCOS; J-PCOS-8: 8-item version of the J-PCOS; SD: standard deviation. ^a^Independent t-test. ^b^The scores range from 1 to 7, with higher scores indicating greater patient care ownership.

## Discussion

In this study, we aimed to develop and validate shorter versions of the J-PCOS. Our findings showed that the J-PCOS can be shortened to versions with 8, 4, or 2 items without significant loss of psychometric properties. Each retained item represented a crucial component of PCO. The use of these shorter versions is expected to improve the feasibility of assessing PCO in research and educational settings.

The development of shorter versions of the J-PCOS is consistent with past research highlighting the importance of concise assessment tools in medical education ^(32), (33)^. Whereas the original 13-item version of the J-PCOS was designed to comprehensively assess PCO ^(10)^, its length posed challenges for practical application, particularly among busy medical residents. This study addresses this challenge by demonstrating that PCO can be assessed with fewer items, potentially resulting in higher response rates and reduced missing data without significant loss of psychometric properties ^(34), (35)^.

We found that the J-PCOS-8 demonstrated better model fit than the J-PCOS. A plausible explanation is that item reduction enhanced construct homogeneity and reduced measurement noise. By retaining the most discriminating and conceptually central items, the shortened scale likely reduced ambiguous wording that introduced error variance in the longer version. Similar findings, wherein shorter versions achieved superior model fit, have been reported in item reduction studies of other scales ^(36), (37)^. Additionally, given their shorter questionnaire length, the internal consistency reliability of the shorter versions appeared similar to that of the J-PCOS. Moreover, all shorter versions could identify significant PCO differences between residents with burnout and those without burnout, as well as between residents with job satisfaction and those without job satisfaction. Thus, our developed measures were well validated as suitable for incorporation into various research and educational settings.

It is important to note that the number of items removed differed across factors, with a greater reduction in the assertiveness domain than in the other domains. This pattern likely reflects how the original J-PCOS was constructed. The assertiveness domain included several items that captured closely related aspects, allowing for the reduction of redundancy without losing key content. In contrast, the other domains included fewer items or items with more distinct meanings, making further reduction less feasible. In developing the shorter versions, we retained at least 1 or 2 items from each factor to preserve the conceptual structure of the original scale. Taken together, the differences in item reduction appear to be consistent with the characteristics of the original scale rather than indicating an imbalance in the shorter versions.

To our knowledge, this is the first study to develop shorter versions of the J-PCOS and examine their psychometric properties. The measures developed in this study have important implications for medical education research and physician training in Japan. First, we anticipate that these measures will be integrated into forthcoming studies in the field of medical education. The 8-item version is expected to be advantageous in applied research settings where reducing respondents’ cognitive burden is important. The 4-item or 2-item versions are particularly well suited to panel studies and large-scale surveys, where time constraints are particularly problematic. Second, given the increasing emphasis on PCO in this era of physician working hour restrictions, these validated tools for evaluating PCO can serve as a valuable metric for assessing training effectiveness. Clinical educators may integrate these shortened scales into competency assessments and curriculum evaluations, allowing for a more systematic approach to monitoring and nurturing PCO. Consequently, these shorter versions have the potential to contribute to the enhancement of educational initiatives and further studies on PCO, which could in turn facilitate more substantial PCO education and ultimately to the delivery of quality patient care.

The findings of our study should be interpreted in light of several potential limitations. First, although we examined the reliability and validity of these shorter versions, we emphasize that this is the first evaluation of these scales. In addition, structural validity was not formally assessed for the 4-item and 2-item versions, as these forms are not suitable for latent variable modeling. Accordingly, our future plan is to verify the psychometric properties of these scales using alternative analytic methods and/or other data. Second, the item selection process mainly relied on subjective judgment by a single expert, which may have limited the breadth of construct representation. Additionally, item selection and validation were conducted using the same dataset, raising the possibility of overfitting. Future research should address these issues by involving multiple experts or using consensus-based approaches to enhance the transparency of item selection and by employing independent samples to confirm the robustness of the findings. Third, the interpretability of the shortened scales has not yet been fully investigated. Although statistically significant differences in mean scores were identified across groups, the practical significance of these differences remains unknown due to the lack of established benchmarks or cutoff values. Future studies should thus elucidate interpretability and determine meaningful thresholds for score differences. Fourth, selection and response biases may have occurred due to the voluntary nature of the online survey. Medical residents with particular characteristics (e.g., a greater interest in professionalism) may have been more likely to participate. Future studies should incorporate approaches such as nonresponse analyses or alternative sampling methods to address potential bias. Fifth, we performed a complete case analysis by excluding participants with missing data, which may have introduced bias if the data were not missing completely at random. For example, if residents with lower levels of PCO or higher burnout were more likely to provide incomplete responses, the observed associations may have been biased. However, the proportion of missing data was relatively small, suggesting that the impact on the overall estimates may be limited. Nevertheless, future studies should consider employing multiple imputation or other appropriate methods to handle missing data and to assess the robustness of the findings. Sixth, the majority of the residents who participated in this study were affiliated with community hospitals. Considering that approximately half of medical residents in Japan train at university main hospitals, the results may have been influenced by this trend. Seventh, the sample in this study exclusively comprised early-stage medical residents (PGY1 and PGY2). Future studies should include physicians at PGY3 or higher. Eighth, a prior sample size estimation was not conducted; instead, we utilized all available cases for this study. Finally, the 2-item version should be interpreted as an ultra-brief indicator and may not fully capture the multidimensional nature of PCO. While it may be beneficial in contexts requiring minimal respondent burden, caution is warranted when employing it as a comprehensive measure of this construct.

### Conclusions

The J-PCOS-8, J-PCOS-4, and J-PCOS-2 were developed and validated. They appear to be reliable and valid measures for PCO that can be as suitably utilized as the longer 13-item version (i.e., the J-PCOS). These shorter versions will likely allow researchers to reduce questionnaire length and in turn achieve higher response rates, improved completion rates, and better data quality. Additionally, they allow clinical educators to use them in daily educational initiatives, which could result in more effective PCO education.

## Article Information

### Acknowledgments

The authors would like to thank the study participants. The authors acknowledge the use of ChatGPT-5.2 (OpenAI) to refine the academic writing.

### Author Contributions

Hirohisa Fujikawa conceived the study with input from Kentaro Sakamaki, Hidetaka Tamune, Yuji Nishizaki, Hiroyuki Kobayashi, and Yasuharu Tokuda. Hirohisa Fujikawa conducted the data analysis with support from Kentaro Sakamaki. Hirohisa Fujikawa drafted the manuscript. All authors discussed, proofread, and approved the final version of the manuscript.

### Conflicts of Interest

Hidetaka Tamune has received honoraria from the Japan Institute for Advancement of Medical Education Program (JAMEP) for preparing examination questions for the General Medicine In-Training Examination (GM-ITE). Yuji Nishizaki has received an honorarium from JAMEP as GM-ITE project manager. Hiroyuki Kobayashi has received honoraria from JAMEP as a speaker at JAMEP lectures. Yasuharu Tokuda is the director of JAMEP and has received an honorarium from JAMEP as a speaker at JAMEP lectures. Hidetaka Tamune, Yuji Nishizaki, Hiroyuki Kobayashi, and Yasuharu Tokuda were not involved in the data analysis. All other authors declare no conflicts of interest.

### Ethics Approval and Participant Consent

This study was conducted in accordance with the Declaration of Helsinki and relevant guidelines. We obtained ethics approval from the ethics committee of the Japan Institute for Advancement of Medical Education Program (approval number 24-10). All participants provided written informed consent by ticking the consent box in the web questionnaire.

## Supplement

Supplementary Material
