# Supplementary material for: Development and Validation of the Shorter Versions of the Patient Care Ownership Scale for Use in Japan
Source: JMA J. 2026 Jul 10;9(5):1104–13. doi: 10.31662/jmaj.2026-0093 (PMC13639660; doi:10.31662/jmaj.2026-0093)
Supplement: Supplementary file 1 [file 2433-3298-9-5-1104-s001.pdf]

Supplementary Table 1. The factor loadings for the items of J-PCOS-8

Factor (aspect)	Item	Factor loadings
Assertiveness (cognitive-behavioral)	Q5. I felt comfortable telling the attending what I felt was the right thing to do for my patients, rather than just letting them decide.	0.63
	Q10. I felt comfortable making decisions independently about my patients' care.	0.85
Sense of ownership (emotional-affective)	Q12. I felt responsible for my patients' care, even after my shift ended.	0.72
	Q13. I felt a strong sense of ownership of my patients' care.	0.85
Diligence (emotional-affective)	Q8. I personally made sure to go back and check that all orders were actually carried out.	0.75
	Q9. When carrying out my patient's management plan, I took extra care to make sure that things did not fall through the cracks.	0.62
Being the "go-to" person (cognitive-behavioral)	Q2. I was the "go-to" person for knowledge about my patients.	0.77
	Q3. I was proactive in checking up on my patients, rather than being called with questions or concerns.	0.57

Abbreviations: J-PCOS-8, 8-item version of the Japanese version of the Patient Care Ownership Scale

Note: Factor loadings are derived from the original validation study of the J-PCOS (Fujikawa H, Son D, Kondo K, et al. Translating and validating a Japanese version of the Patient Care Ownership Scale: a multicenter cross-sectional study. BMC Med Educ. 2021;21(1):415.). Detailed factor analytic results are reported in the original publication.

Supplementary Item S1. 8-item version of the Japanese version of the Patient Care Ownership Scale

以下の質問文では、あなたが入院患者に関する業務を行った過去 2〜4 週間、どのように感じていたのかについておたずねします。それぞれの質問に対して、どの程度賛成あるいは反対かを、以下の選択肢の中から選んでください。ただし、あなたが本研究参加時点で従事している業務のみに基づいて、回答してください。

Q1. 私は、担当患者について誰よりもよく知っていると周りに頼りにされました。

1 □ 強く反対する 2 □ 反対する 3 □ いくらか反対する 4 □ どちらでもない
5 □ いくらか賛成する 6 □ 賛成する 7 □ 強く賛成する

Q2. 私は、質問や懸念について呼びかけられるよりもむしろ、先回りをして担当患者について確認していました。

1 □ 強く反対する 2 □ 反対する 3 □ いくらか反対する 4 □ どちらでもない
5 □ いくらか賛成する 6 □ 賛成する 7 □ 強く賛成する

Q3. 私は、担当患者の診療方針について、上級医まかせにするのではなく、患者にとって最善だと私が思ったことを上級医に対して気兼ねなく伝えました。

1 □ 強く反対する 2 □ 反対する 3 □ いくらか反対する 4 □ どちらでもない
5 □ いくらか賛成する 6 □ 賛成する 7 □ 強く賛成する

Q4. 私は必ず、すべての指示が実際に行われたかどうか、さかのぼって自ら確認しました。

1 □ 強く反対する 2 □ 反対する 3 □ いくらか反対する 4 □ どちらでもない
5 □ いくらか賛成する 6 □ 賛成する 7 □ 強く賛成する

Q5. 私は、担当患者の診療計画を実行する際、万が一なにか抜け落ちていることがないか確認するため十分に注意を払いました。

1 □ 強く反対する 2 □ 反対する 3 □ いくらか反対する 4 □ どちらでもない
5 □ いくらか賛成する 6 □ 賛成する 7 □ 強く賛成する

Q6. 私は気兼ねなく、担当患者のケアについて自主的に決断を下しました。

1 □ 強く反対する 2 □ 反対する 3 □ いくらか反対する 4 □ どちらでもない
5 □ いくらか賛成する 6 □ 賛成する 7 □ 強く賛成する

Q7. 私は、自分の勤務シフトの終了後も、担当患者のケアに関して責任を感じていました。

1 □ 強く反対する 2 □ 反対する 3 □ いくらか反対する 4 □ どちらでもない
5 □ いくらか賛成する 6 □ 賛成する 7 □ 強く賛成する

Q8. 私は、担当患者のケアに対して強い当事者意識を持っていました。

1 □ 強く反対する 2 □ 反対する 3 □ いくらか反対する 4 □ どちらでもない
5 □ いくらか賛成する 6 □ 賛成する 7 □ 強く賛成する

※ 本尺度使用時は、責任著者にメールで許諾を得たうえで、本論文の引用をお願いいたします。

Supplementary Item S2. 4-item version of the Japanese version of the Patient Care Ownership Scale

以下の質問文では、あなたが入院患者に関する業務を行った過去 2〜4 週間、どのように感じていたのかについておたずねします。それぞれの質問に対して、どの程度賛成あるいは反対かを、以下の選択肢の中から選んでください。ただし、あなたが本研究参加時点で従事している業務のみに基づいて、回答してください。

Q1. 私は、担当患者について誰よりもよく知っていると周りに頼りにされました。

1 □ 強く反対する 2 □ 反対する 3 □ いくらか反対する 4 □ どちらでもない
5 □ いくらか賛成する 6 □ 賛成する 7 □ 強く賛成する

Q2. 私は必ず、すべての指示が実際に行われたかどうか、さかのぼって自ら確認しました。

1 □ 強く反対する 2 □ 反対する 3 □ いくらか反対する 4 □ どちらでもない
5 □ いくらか賛成する 6 □ 賛成する 7 □ 強く賛成する

Q3. 私は気兼ねなく、担当患者のケアについて自主的に決断を下しました。

1 □ 強く反対する 2 □ 反対する 3 □ いくらか反対する 4 □ どちらでもない
5 □ いくらか賛成する 6 □ 賛成する 7 □ 強く賛成する

Q4. 私は、担当患者のケアに対して強い当事者意識を持っていました。

1 □ 強く反対する 2 □ 反対する 3 □ いくらか反対する 4 □ どちらでもない
5 □ いくらか賛成する 6 □ 賛成する 7 □ 強く賛成する

※ 本尺度使用時は、責任著者にメールで許諾を得たうえで、本論文の引用をお願いいたします。

Supplementary Item S3. 2-item version of the Japanese version of the Patient Care Ownership Scale

以下の質問文では、あなたが入院患者に関する業務を行った過去 2〜4 週間、どのように感じていたのかについておたずねします。それぞれの質問に対して、どの程度賛成あるいは反対かを、以下の選択肢の中から選んでください。ただし、あなたが本研究参加時点で従事している業務のみに基づいて、回答してください。

Q1. 私は気兼ねなく、担当患者のケアについて自主的に決断を下しました。

1 □ 強く反対する 2 □ 反対する 3 □ いくらか反対する 4 □ どちらでもない
5 □ いくらか賛成する 6 □ 賛成する 7 □ 強く賛成する

Q2. 私は、担当患者のケアに対して強い当事者意識を持っていました。

1 □ 強く反対する 2 □ 反対する 3 □ いくらか反対する 4 □ どちらでもない
5 □ いくらか賛成する 6 □ 賛成する 7 □ 強く賛成する

※ 本尺度使用時は、責任著者にメールで許諾を得たうえで、本論文の引用をお願いいたします。
